# Brainstem Auditory Evoked Potentials in Raccoon Dogs (*Nyctereutes procynoides*)

**DOI:** 10.3390/ani10020233

**Published:** 2020-02-02

**Authors:** Raluca Ștefănescu, Constantin Roman, Liviu Dan Miron, Gheorghe Solcan, Vasile Vulpe, Luminița Diana Hrițcu, Mihai Musteata

**Affiliations:** 1 Neurology Clinical Unit, Clinics Department, Faculty of Veterinary Medicine, University of Agricultural Sciences and Veterinary Medicine, Ion Ionescu de la Brad, 700489 Iasi, Romania; raluca.stef@yahoo.ro; 2 Parasitology Service, Clinics Department, Faculty of Veterinary Medicine, University of Agricultural Sciences and Veterinary Medicine, Ion Ionescu de la Brad, 700489 Iasi, Romania; roman.const@yahoo.com (C.R.); lmiron@uaiasi.ro (L.D.M.); 3 Internal Medicine Unit, Clinics Department, Faculty of Veterinary Medicine, University of Agricultural Sciences and Veterinary Medicine, Ion Ionescu de la Brad, 700489 Iasi, Romania; gsolcan@uaiasi.ro; 4 Radiology Unit, Clinics Department, Faculty of Veterinary Medicine, University of Agricultural Sciences and Veterinary Medicine, Ion Ionescu de la Brad, 700489 Iasi, Romania; vvulpe@uaiasi.ro

**Keywords:** brainstem auditory evoked response, BAER, latencies, raccoon dog

## Abstract

**Simple Summary:**

Raccoon dogs (*Nyctereutes procynoides*) are canids indigenous to Eastern Asia considered being one of the most invasive non-native animals in Europe. Due to the lack of natural enemies, the spread of the species into the Europe areal raises the possibility of the spread of some hazardous zoonoses for which is a vector including viruses able to cause fatal encephalitis (e.g., rabies). In this light, from a clinical perspective, objective, quick, cost-effective techniques for investigating the integrity and functionality of the nervous system are needed. Our study investigates for the first time the auditory system function of the raccoon dogs by using brainstem auditory evoked technique. Raccoon dogs share similarities with other species regarding brainstem auditory evoked potentials (responses) (BAER) morphology. The obtained traces where homogenous according to the latencies and amplitudes of the waves and may be used for further studies regarding the nervous system physiology in this species but also to help clinicians to asses any functional impairment of auditory system in this species.

**Abstract:**

Raccoon dogs (*Nyctereutes procynoides*) are canids indigenous to Eastern Asia being one of the most invasive non-native animals in Europe and potential vectors for several hazardous parasitic and viral diseases. To present, there is a lack of studies regarding objective techniques used to appreciate the integrity and functionality of the nervous system in this species. Brainstem auditory evoked potentials (BAER) is a cost-effective, quick and noninvasive technique to assess the functionality of nervous system. The aim of the study is to provide reference values and evaluates the reliability of BAER recording with needle electrodes in clinically healthy raccoon dogs. Nine individuals were investigated for BAER under xylazine and ketamine general anesthesia. Four waves (I, II, III and V) were identified and evaluated for latencies, morphologically similarity to those previously reported for other species (dogs, horses, cats, and ferret). Our data can be used in further studies to asses objectively the auditory system function in raccoon dogs.

## 1. Introduction

Raccoon dogs (*Nyctereutes procynoides*) are canids indigenous to Eastern Asia but they have extended their range into Eastern Europe during the last decades [1,2]. Raccoon dog acclimatized very well because of its eating habits (omnivorous predator), reproductive capacity and lack of natural enemies. The species is regarded as one of the most invasive non-native animals in Europe. Raccoon dogs are considered potential vectors for several hazardous parasitic and viral diseases such as rabies and canine distemper [3]. To present, there is a lack of studies regarding objective techniques used to appreciate the integrity and functionality of the nervous system in raccoon dogs. Moreover, most of the techniques used in nervous system assessment are expensive (e.g., advanced imaging) or invasive (cerebrospinal fluid analysis). Brainstem auditory evoked response technique (BAER) is performed for evaluating the auditory function (audiogram) and it is used to help identify conductive and sensorineural deafness. In patients for whom the auditive impairment is the only deficit, performing the audiogram is desirable. However, from a clinical point of view, especially in clinical neurology, BAER technique is easy to discriminate against the neurolocalization between central and peripheral components of the auditory system in patients with vestibular signs. That may be important especially in patients with brainstem impairment (e.g., encephalitis) or non-equivocal symptomatology (central versus peripheral component) but who need a rapid, less expensive and objective orientation in diagnostic procedures. The technique is cost-effective, quick and noninvasive. Audiogram testing uses different intensities for the auditive stimulus. For neurolocalization purposes, a standard intensity (e.g., 90 dB sound pressure level (SPL) [4,5,6,7]) is preferred. To present, in the absence of reference values, the electrical activity of these structures can only be interpreted by comparison with values obtained contralaterally. To the best of our knowledge, there is no report regarding BAER testing in raccoon dogs.

The aim of the study is to identify the reference values and evaluates the reliability of BAER recording with needle electrodes in clinically healthy raccoon dogs when a standard intensity stimulus (90 dB SPL) is applied.

## 2. Materials and Methods

The study was carried out in the Neurology Department of the Veterinary Teaching Hospital (VTH) of the Faculty of Veterinary Medicine from Iasi, Romania. Ethical approval for the study was obtained from the Ethics Committee of the Faculty of Veterinary Medicine, University of Agricultural Sciences and Veterinary Medicine “Ion Ionescu de la Brad” from Iași (no. 429/05.05.2018).

### 2.1. Animals

The study was performed on nine clinically healthy individuals (six males, three females) aged between 1 and 3 years, weighing 5 to 6.6 kg (average weight 5.43 kg), with no neurological or known hearing disorders. All individuals where captured and bred in captivity in pens placed in a forest environment.

### 2.2. BAER System and Electrodes

BAER examinations were made using Neuropack S, MEB 9400K electrodiagnostic system (Nihon Kohden, Tokyo, Japan) in the auditory brainstem response program (ABR). All the examinations were performed after the initial calibration of the equipment according to the manufacturer recommending. Electrical signals were captured using subcutaneous steel needle electrodes (12 × 0.35 mm, 1mm pin) placed as follows: the recording electrode was inserted over the vertex, the negative electrodes near the mastoid prominence of each ear and the ground electrode on the median line, retro-ocipitally. Impedance was kept below 5 Ω and alternating 0.1 ms click stimuli were applied through foam earphones inserted in each auditory canal. Stimulus intensity was set at 90 dB SPL; in monaural recording, the non-tested ear was masked by white noise with an intensity of 50 dB SPL to eliminate the crossover effect [6,7,8,9]. Each waveform resulted was an average of 1000 stimulations. The broadband click included frequencies between 100 (low cut) and 3000 Hz (high cut). Latencies were manually marked, each positive peak being labeled in sequence by Roman numerals from I to V. Four waves were obtained, their latencies (the time between the auditory stimulus and the peak expressed in milliseconds (ms)) were measured, as well as the intervals I–III, III–V, I–V. As long as the investigated group was represented by healthy individuals without a history of known neurological or hearing impairment, the Ethics Committee did not approve multiple general anesthesias. Therefore, to assure repeatability, two BAER examinations were taken for each raccoon dog at a 5 min interval during one single general anesthesia. The final value represents the mean of those two examinations.

### 2.3. Experiment Protocol

The recording was carried out in a quiet room, under sedation with xylazine (60 μg/kg i.m.) and ketamine (80 μg/kg i.m.). All subjects were deeply sedated for at least 20 min period during which the BAER testing was performed. Before the BAER examination, the external canals of both ears were examined by using a video otoscope (UB CAM Pro Videoscope, KRUUSE, Langeskov, Denmark) in order to exclude external otitis or cerumen accumulation. Then the raccoon dogs were positioned in sternal recumbency and their oxygen level was monitored by a pulse oximeter.

### 2.4. Statistical Analysis

The statistical interpretation of the results was made with the software Statistical Package for the Social Sciences for Windows (SPSS) 20 (IBM Corp., Armonik, NY, USA), using the tests Kruskall Wallis, Man–Whitney U and Wilcoxon Signed Ranks Test for 2 paired samples, with a significance threshold *p* < 0.05.

## 3. Results

All the animals recovered from sedation in less than 60 min and no cardiac or respiratory events were noticed during the procedure time. BAER examination was successful for all individuals, thus, for each raccoon dog, a pair of mono and binaural BAER traces were further analyzed.

**Figure 1:**
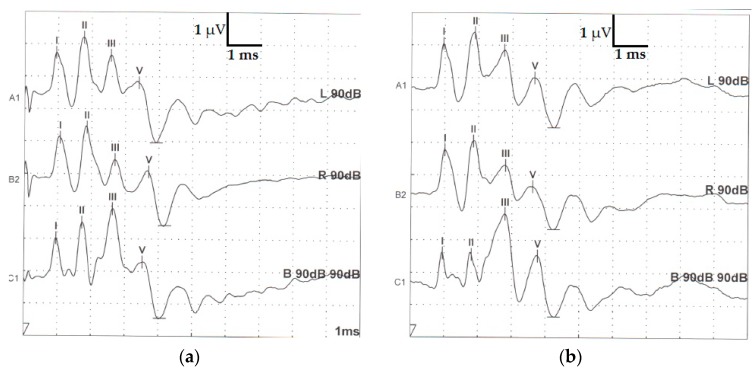
Typical morphology of two adult healthy raccoon dog brainstem auditory evoked responses recorded ((**a**) individual 7 and (**b**) individual 9) after stimulation of the left ear (upper trace), right ear (middle trace) and binaural stimulation (bottom) under general anesthesia. (horizontal bar—ms; vertical bar µV—microvolts).

After visual inspection of registered traces, four waves (I, II, III and V) were identified in all raccoon dogs’ recordings (Figure 1).

In all traces, the second wave (II) was positive and had the greatest amplitude (the voltage difference between the positive peak and the negative peak. Wave V was recorded as the positive peak occurring immediately before a deeply negative trough occurring in the second half of the recording. According to the marking methodology, we assumed that wave IV was absent in any of the records. The values obtained for waves’ latency and interwave latency (I–III, III–V, I–V) in monaural and binaural stimulation are presented in Table 1 and Table 2. Amplitude was calculated for each wave and further analyzed (Table 3).

In most of the binaural traces, wave I appeared morphologically to be composed of two peaks (see Figure 1).

No statistical differences (*p* > 0.05) were observed between the first and second stimulation sessions for each individual (e.g., *p* ≥ 0.817 for latencies, *p* ≥ 0.058 for amplitudes), therefore the repeatability of the experimental procedure was assured over a short time scale. The homogeneity of the group was tested by comparing both the latencies and amplitudes values between individuals or gender and no significant differences were noticed (*p* = 0.433). Furthermore, no significant differences were observed when the wave’s latency values were compared after the right, left ear or binaural stimulation (*p* ≥ 0.661 for latencies) for each individual. When compared the amplitudes values (left vs. right, left and right vs. binaural stimulation) no statistical differences were obtained in all tested groups (*p* ≥ 0.219).

**Figure 2:**
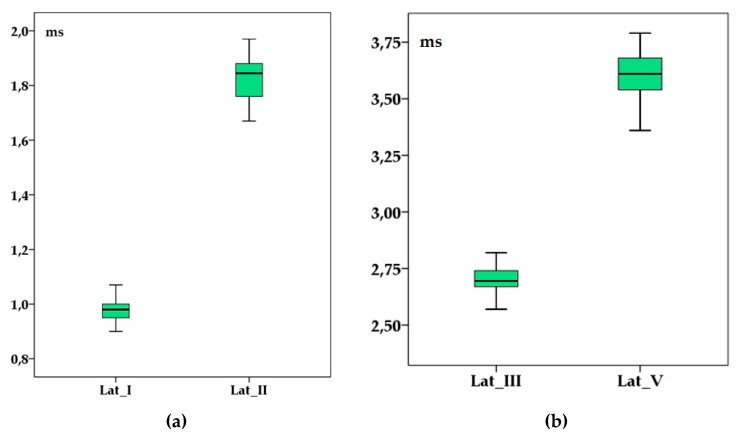
Brainstem auditory evoked response’ latencies (in milliseconds) of wave I and II (**a**) and III and V (**b**) in adult healthy raccoon dogs after a 90 dB sound pressure level (SPL) stimulus was applied. Boxplots represent the cumulative values from all individuals after two consecutive left, right and binaural stimulations. Lat_I—wave I latency; Lat_II—wave II latency; Lat_III—wave III latency; Lat_V—wave V latency.

**Figure 3:**
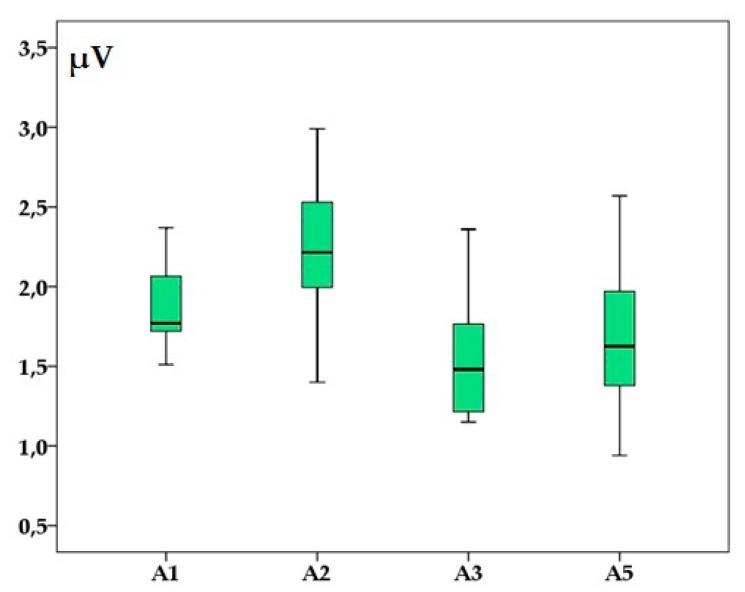
Brainstem auditory evoked response’ amplitudes (in microvolts) of waves I (A1), II (A2), III (A3) and V (A5) in adult healthy raccoon dogs after a 90 dB SPL stimulus was applied. Boxplots represent the cumulative values from all individuals after two consecutive left, right and binaural stimulations.

The entire group means latencies and amplitudes’s values are presented in Figure 2 and Figure 3.

## 4. Discussion

In this paper, we describe the brainstem evoked potentials in raccoon dogs. To the best of our knowledge, this is the first study in which the function of auditory nuclei was assessed by the BAER technique on this species. Thus, to identify the waves we used the methodology used for dogs and cats for which the first positive peak is representing wave I and a positive peak occurring immediately before the deeply negative trough in the second half of the recording is represented by the wave V [10,11]. Wave IV is often absent in dogs and inconstant in cats (in which it can make a complex together with wave V) [11]. By working with the methodology used for dogs and cats and not the one for humans we avoided thus the mislabeling of wave V.

In our individuals, waves’ morphology (wave I, II, III and V) (Figure 1) was similar to the one described in dogs, cats, and other mammals [6,7,8,12,13]. In ferrets, Piazza et al. (2014) observed that peak III consists of two peaks but that does not seem to be the case in our individuals. We can assume that raccoon dogs can share the same auditory nuclei anatomical locations and physiological responses. Despite the fact that some discrepancies and uncertainties still persist over the nuclear generators of the waves, it is generally accepted that peak I is produced by stimulation of the extra-cranial part of the auditory nerve [4,14,15,16,17] and that peak II is presumably generated by the ipsilateral cochlear nucleus [7,16]. Peaks’ III to V neuro-anatomical origin variability (olivary nuclei, lateral lemniscus, and caudal colliculi) have been reported [7,11,15].

When the auditive stimulus is applied to an ear, a crossover recording (crossover effect) from a functional contralateral ear can be obtained. This effect was described both in humans and animals [9,18] and in order to eliminate it, white noise is delivered in the non-tested ear. In a study performed on fifty-six deaf Dalmatian dogs [9], when a contralateral white stimulus with 20 dB below the tested ear was applied, the authors observed the abolition of BAER wave V in the deaf ear suggesting that this wave was produced as an effect of crossover effect. In humans, the non-tested ear is usually masked for a better analysis of BAER waves, especially when substantial differences are observed between the left and right ear. In veterinary patients, the contralateral ear masking is not a requirement but is recommended to be used especially when unilateral deafness is suspected. It was reported that the minimum intensity used to abolish the contralateral effect in dogs is 20 dB lower than the tested ear [9]. However, this study used a different unit for sound intensity (dB HL). When BAER testing was performed using the dB SPL intensity scale, white noise with 50 dB SPL intensity applied on the contralateral ear will abolish the crossover effect [4,7].

In the present study, the observed values of the wave latencies and intervals are lower than those described previously in other species like dogs (e.g., 1.098 ± 0.025 ms for latency I and 3.827 ± 0.226 ms for latency V [6] or 1.59 ± 0.1 ms for latency I and 5.56 ± 0.19 ms for latency V [19]) and ferrets where a value of 1.1 ± 0.09 ms for wave I latency and 3.8 ± 0.21 ms for wave V latency was reported [4]. Regarding the amplitudes, their values were similar or smaller than in other reports. For example, wave I amplitude was 1.6 ± 0.36 µV in ferrets [4] and 2.356 ± 0.463 in dogs [6]. However, in clinical conditions, the amplitudes are usually assessed by comparison with the contralateral ones than accordingly with a specific reference value. The intensity of the stimulus used to induce BAER has varied in previously reported studies but in the present study, an intensity of 90 dB SPL was chosen for stimulation as this is the standard value used in dogs, cats [11,19] and ferrets [4]. At lower intensity of the stimulus, the wave’s amplitude is expected to decrease simultaneously with an increase of the latencies. Individual amplitude variability is often observed in different species and the assessment of this parameter is done usually by comparing with the contralateral ear waves. However, in our subjects, no differences were observed when the amplitudes were investigated. In our study, the broadband click used included frequencies between 100 (low cut) and 3000 Hz (high cut). As for the latencies, with respect to the clinical standard, we used the methodology reported in dogs or cats [7,9]. A shorter high cut (e.g. 1500 Hz) may be used eventually if the traces are not smooth enough for interpretation but anyway this was not the case in this study. In our case, the recordings were made under general anesthesia by using xylazine and ketamine, assuming that this method is as suitable as for dogs [20], cats [6] and gerbils [21]. Both xylazine and ketamine appears to have no effect over the registered BAER latency or waveform amplitudes registered [12]. However, Sims and Horohov [22] found out that in cats, the latencies for waves III/IV and V were increased when the individuals were under the effect of xylazine and ketamine but in this report, the applied stimulus was 90 dB HL not 90 dB SPL.

Raccoon dogs have a maximum life span of 7–8 years and reproductive maturity is reached before 1 year of age [23]. In our study, all the studied animals were aged between 1 and 3 years being considered as young adults. In this way, we eliminate the influence of both incomplete development of the auditory system and geriatric influence over its activity. Full maturation of BAER in dogs and cats occurs by 40 days [10,15]. In ferrets, adult BAER waveform is achieved between 34 and 40 days of age [10,15,17]. However, the raccoon dogs’ postnatal brainstem maturation needs further studies. In consequence, our results are not necessarily applicable to younger individuals in which the nervous system is not fully developed.

In raccoon dogs, the skull is small and moderately elongated, without an evident sexual dimorphism (excepting the mandible bone) [24] sharing similarities with mesocephalic dogs. Our results sustain the previously reported data in dogs in which the head size did not influence the latencies and amplitudes of BAER waves [25].

Altough in the present study, we obtained a great similarity with dogs, cats or ferret’s BAER with little differences in the latencies of waves, we agreed on some limitations. Firstly, the number of analyzed individuals is low. We cannot exclude that some inter-individual differences will occur in larger groups. Secondly, the BAER was measured to a single stimulus at a single intensity, according to the clinical environment. Further studies in which the hearing range in raccoon dogs is investigated are needed. By performing an audiogram, differences between raccoon dogs and other species (dogs, cats, ferrets, etc.) might be observed and consequently used to create a particular standardization of BAER parameters for raccoon dogs’ examination. In the same line, different frequencies of the broadband click might be used. This would also yield information useful in a biological context. Thirdly, in our study, the anesthesia was made with xylazine and ketamine. Our results are not necessarily applicable when a different anesthetic drug (or combination) is used. Finally, due to ethical considerations, our data are obtained after two stimulations under one general anesthesia episode. Hence, further studies with repeated examinations of the same animals but across slight differences in electrode placement and over larger time intervals are needed to observe the appearance of any latencies’ or amplitudes’ intra/inter-individual variability.

## 5. Conclusions

In this paper, we describe for the first time the values of latencies and amplitude of BAER waves and evaluate the reliability of BAER recording with needle electrodes in clinically adult healthy raccoon dogs. Despite the limited number of animals included in this study, the obtained traces are morphologically similar to those previously reported for other species (dogs, cats, ferrets). Our data can be used in further studies to asses objectively the auditory system function in raccoon dogs.

## Figures and Tables

**Table 1:** Brainstem auditory evoked potentials latencies for waves I, II, III and V (in ms) in healthy adult anesthetized raccoon dogs.

Latency (ms)	Left Ear	Right Ear	Binaural
I	0.95 ± 0.03	1.00 ± 0.03	0.99 ± 0.08
II	1.84 ± 0.05	1.83 ± 0.12	1.79 ± 0.08
III	2.70 ± 0.07	2.73 ± 0.12	2.70 ± 0.05
V	3.54 ± 0.10	3.61 ± 0.08	3.65 ± 0.09

**Table 2:** Brainstem auditory evoked potentials interwave I–III, III–V and I–V (in ms) in healthy adult anesthetized raccoon dogs.

Interwave	Left Ear	Right Ear	Binaural
Interwave I–III	1.74 ± 0.05	1.72 ± 0.14	1.73 ± 0.09
Interwave III–V	0.84 ± 0.06	0.84 ± 0.06	0.94 ± 0.06
Interwave I–V	2.58 ± 0.09	2.60 ± 0.09	2.68 ± 0.11

**Table 3:** Brainstem auditory evoked potentials amplitudes for waves I, II, III and V (in µV) in healthy adult anesthetized raccoon dogs.

Amplitude (µV)	Left Ear	Right Ear	Binaural
I	1.82 ± 0.49	1.92 ± 0.29	1.63 ± 0.68
II	2.33 ± 0.64	2.33 ± 0.40	2.01 ± 1.17
III	1.57 ± 0.34	1.51 ± 0.33	2.76 ± 0.51
V	1.72 ± 0.41	1.63 ± 0.41	2.25 ± 0.60
